# 

**Published:** 1896-01

**Authors:** 


### CONTENTS.

ORIGINAL ARTICLES:
- Intestinal Obstruction in the Dog. By CECIL FRENCH, D.V.S. . . . . . 1
- The Horse as a Producer of Antitoxins. By OLOF SCHWARZKOPF, V.M.D. . . 13
- Serum-therapeutics. By S. J. J. HARGER, V.M.D. . . . . . . . 18
- Address to the Association of Veterinary Faculties of North America. By J. H. WATTLES, V.S. . . . . . . . . . . . 23
- Tetanus. By S. H. KINGERY . . . . . . . . . . 27

REPORTS OF CASES:
- A Case of Tetanus Cured with Antitoxin. By GEORGE JOBSON, V.S. . . . 33
- Influence of Fever on a Case of Traumatic Tetanus. By A. S. WHEELER, V.M.D. 35

Extracts from Foreign Journals . . . . . . . . . . 37

EDITORIALS:
- "Journal" Change . . . . . . . . . . 47
- Buffalo Wins for 1896 . . . . . . . . . . 47
- Rank for our Army Veterinarians . . . . . . . . 48
- To Our Readers . . . . . . . . . . 49
- Appointment of State Veterinarian for Pennsylvania . . . . . 50

Obituary—Married . . . . . . . . . . 50

LEGISLATION—Maryland . . . . . . . . . . 51

REVIEWS . . . . . . . . . . 51

U. S V. M. A. . . . . . . . . . . 52

CONTROL WORK . . . . . . . . . . 53

AMONG THE COLLEGES . . . . . . . . . . 54

SOCIETY PROCEEDINGS:
- Iowa State Veterinary Medical Association . . . . . . . 58
- Abstract of Stenographer's Report, U. S. V. M. A., 1895 . . . . . 67
- Illinois State Veterinary Medical Association . . . . . . 70
- Western Iowa Veterinary Medical Association . . . . . . 70
- Connecticut Veterinary Medical Association . . . . . . 71
- Keystone Veterinary Medical Association . . . . . . 71
- Schuylkill Valley Veterinary Medical Association . . . . . 72
- Ontario Veterinary Medical College Society . . . . . . 73
- Wisconsin Society of Veterinary Graduates . . . . . . 73
- McGill University Proceedings for the Year 1894–95 . . . . . 73
- New York County Veterinary Medical Association . . . . . 78

PERSONAL . . . . . . . . . . 79

OF PASSING INTEREST . . . . . . . . . . 81

NEW INVENTIONS . . . . . . . . . . 83

